# *BETMB*: A Dual‐Target Compound for Synergistic Suppression of Neuronal Hyperexcitability in Refractory Epilepsy via Concurrent Modulation of Nav Channels and *GABA_A_* Receptors

**DOI:** 10.1002/cns.70766

**Published:** 2026-01-23

**Authors:** Di Zhang, Kai Li, Yingying Zhang, Yao Nie, Yue Li, Rui Li, Xin Wang, Shengjun Mao

**Affiliations:** ^1^ Key Laboratory of Drug‐Targeting and Drug Delivery System of the Education Ministry and Sichuan Province, Sichuan Engineering Laboratory for Plant‐Sourced Drug and Sichuan Research Center for Drug Precision Industrial Technology, West China School of Pharmacy Sichuan University Chengdu Sichuan China

**Keywords:** antiseizure medication, epilepsy, GABA_A_ receptor, synergistic effect, voltage‐gated sodium channel

## Abstract

**Aims:**

This study aims to evaluate 5‐(but‐1‐en‐1‐yl)‐1,2,3‐trimethoxybenzene (BETMB) as a novel dual‐target anti‐seizure agent for refractory epilepsy and elucidate the synergistic neuroelectrophysiological mechanism between Na_V_ channels and GABA_A_ receptors.

**Methods:**

Whole‐cell patch‐clamp recordings characterized BETMB's dual‐target activity. Antiseizure efficacy was assessed in maximal electroshock (MES), pentylenetetrazole (PTZ), and kainic acid (KA) models. Cognitive function in chronic KA mice was evaluated using the Morris water maze (MWM). Histopathological, immunohistochemical, and Western blot analyses explored neuroprotection. Synergy between Na_V_ and GABA_A_R modulation was systematically investigated using both an in vitro Mg^2+^‐free model of neuronal hyperexcitability and an *in silico* model of cortical spreading depolarization (CSD).

**Results:**

BETMB acted as a GABA_A_R positive allosteric modulator (EC_50_ = 93.2 μM) and a state‐dependent Na_V_ blocker (K_I_ = 1.9 μM). It significantly suppressed seizures across models, improved cognition in chronic epilepsy, and modulated downstream expression of GABRA1, NR2B, and BDNF‐pAKT‐CREB signaling. Synergistic Na_V_ and GABA_A_R modulation completely abolished ictal‐like discharges in Mg^2+^‐free cellular models and prevented CSD initiation in computational simulations.

**Conclusion:**

BETMB is a promising dual‐target therapy for refractory epilepsy, supported by the first electrophysiological evidence that dual modulation of GABA_A_R and Na_V_ synergistically suppresses neuronal hyperexcitability. Beyond epilepsy, this finding may also extend to CSD‐related conditions such as stroke, traumatic brain injury, and migraine.

## Graphical abstract

BETMB exerts multi‐target effects by positively modulating GABA_A_Rs and blocking Na_V_ channels in a state‐dependent manner. This suppresses neuronal hyperexcitability, leading to seizure control and restoration of cognitive function.

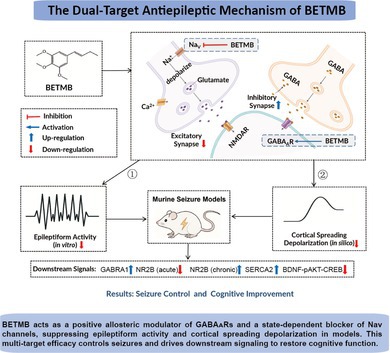

## 1 Introduction

Epilepsy, a chronic neurological disorder affecting approximately 50 million people worldwide, is characterized by recurrent unprovoked seizures caused by abnormal neuronal hyperexcitability. Although current antiseizure medications (ASMs) effectively control seizures in about 70% of patients, approximately 30% develop refractory epilepsy, defined as continued seizure activity despite optimized pharmacological treatment, leading to increased mortality risks and substantial impairments in quality of life [1, 2].

The persistent 30% prevalence of drug‐resistant epilepsy (DRE), despite three decades of advancements in ASM development, highlights significant therapeutic challenges. Epileptogenesis encompasses a range of complex mechanisms, such as ion channel dysfunction, synaptic remodeling, and network hypersynchronization. Most modern ASMs act through three primary mechanisms: modulation of voltage‐gated sodium and calcium channels, enhancement of GABAergic neurotransmission, and inhibition of glutamatergic signaling. Although monotherapy remains the first‐line treatment strategy, approximately 30% of patients ultimately require combination therapy after failing sequential monotherapies [3]. Current clinical guidelines recommend considering polytherapy after two appropriately selected ASMs prove ineffective, thereby establishing a therapeutic paradigm that is based on mechanistic rationale rather than empirical selection [4].

With more than twenty ASMs available in clinical practice, the number of potential dual‐drug regimens reaches into the hundreds, and triple‐drug combinations could amount to thousands. Although many of these combinations may not be clinically feasible, numerous effective regimens remain unexplored due to the inherent complexity. Therefore, a more focused approach to the treatment of DRE is essential [5]. Mechanism‐driven strategies that are based on pharmacological synergy, rather than empirical trial‐and‐error approaches, are urgently needed. Current knowledge of the mechanisms of ASMs, *including* ion channel modulation and neurotransmitter regulation, provides a rational foundation for such strategies. However, significant gaps remain in our understanding of epileptogenic network pathophysiology and individual variations in pharmacodynamic responses that contribute to treatment resistance [6]. These gaps underscore the necessity for further advancements in neuroelectrophysiological and neurobiological research to optimize therapeutic interventions.

Rational polytherapy necessitates a thorough understanding of the mechanistic interactions among ASMs. Historically, combinations such as phenobarbital, phenytoin, and carbamazepine were developed empirically before their underlying mechanisms were elucidated [7], yet they demonstrated enhanced efficacy through pathways that remain largely unclear. Current evidence suggests synergistic effects may occur when sodium channel blockers (e.g., carbamazepine) are combined with GABA_A_R modulators (e.g., clonazepam), achieving superior seizure control in patients with refractory epilepsy [4, 8]. There is a strong synergistic effect between valproic acid (which acts through multiple mechanisms including Na_V_ blockade and GABA enhancement) and lamotrigine (Na_V_ blocker) in the treatment of both focal‐onset and generalized seizures. In a study conducted by Brodie and Yuen [9], lamotrigine monotherapy was substituted in patients who were not adequately controlled by carbamazepine, phenytoin, or valproic acid. Initially, all patients were stabilized using combination therapy, with lamotrigine doses adjusted according to known pharmacokinetic interactions. Plasma lamotrigine concentrations were comparable across all treatment groups. The responder rate was significantly higher in the valproic acid group than in the groups receiving lamotrigine with either carbamazepine or phenytoin. This clinical evidence, further supported by preclinical studies that quantify synergistic indices in animal models, strongly indicates that the therapeutic enhancement goes beyond pharmacokinetic interactions and involves complementary pharmacodynamic mechanisms, including the dual modulation of Na_V_s and GABAergic transmission [10].

Inspired by the clinical and preclinical rationale for synergistic dual‐target therapy, we investigated 5‐But‐1‐enyl‐1,2,3‐trimethoxybenzene (BETMB), a compound derived from structural optimization of α‐asarone by Zhang et al. [11]. Our study aims to validate BETMB as a single chemical entity designed to concurrently engage both Na_V_ channels and GABA_A_ receptors, and to provide a comprehensive electrophysiological and molecular characterization of its anti‐seizure effects.

## 2 Methods

### 2.1 Reagents

BETMB was synthesized according to a reference method *[11]*, with a purity of 99%. All chemicals were of cell culture grade purity.

### 2.2 Cell Culture

Primary cortical neurons from newborn Sprague–Dawley rats (P0) were prepared and cultured as previously described [12]. In brief, neurons were dissociated and cultured in Neurobasal‐A medium supplemented with B‐27 on poly‐L‐lysine‐coated coverslips. Electrophysiological experiments were conducted on days 10*–*14 in vitro.

### 2.3 Patch‐Clamp Recordings

Whole‐cell patch‐clamp recordings were employed to characterize BETMB's pharmacological properties through three experimental approaches: (1) modulation of GABA_A_ receptors in isolated cultured neurons, (2) inhibition of Na_V_ channels in isolated neurons, and (3) validation of the synergistic interaction between Na_V_ blockade and GABA_A_R potentiation in established neuronal networks using the Mg^2+^‐free model of network hyperactivity and hypersynchrony. Detailed recording solutions, specific voltage protocols, drug application procedures, and comprehensive analytical methods are provided in the Supporting Information.

### 2.4 Animals and Ethical Issues

Animals were provided by Chengdu Dossy Experimental Animals Co. Ltd. All animal experimental procedures were ethically approved by the Sichuan University Ethics Committee for the Use of Laboratory Animals with a license number SYXK (Chuan) 2018–113. All animal experiments were conducted in accordance with the ARRIVE guidelines and the National Institutes of Health guidelines for laboratory animal care.

### 2.5 Pharmacokinetic and Tissue Distribution Studies

For pharmacokinetic and tissue distribution studies, male Sprague–Dawley rats (200 ± 20 g) were used. After acclimatization and fasting, rats received a single dose of BETMB (50 mg/kg) via intravenous (i.v.) or intraperitoneal (i.p.) injection. For pharmacokinetics, serial blood samples were collected from the retro‐orbital plexus at predefined time points (3 min to 10 h). For tissue distribution, rats were euthanized at 5, 15, and 30 min post i.p. administration, and major organs (brain, heart, liver, spleen, lung, kidney) were harvested. Plasma and tissue homogenate supernatants were obtained by centrifugation and stored at −80°C for HPLC analysis.

### 2.6 Animal Epilepsy Models

Male C57BL/6 mice were used in acute (MES, PTZ, KA) and chronic (KA) epilepsy models. Animals were randomized into groups receiving vehicle, BETMB (50, 75, 100 mg/kg), or positive controls. Seizure severity (Racine scale) and latency were assessed. Chronic mice were treated for 20 days before Morris water maze (MWM) testing. Detailed protocols are in the Supporting Information.

### 2.7 MWM Test

Spatial learning and memory were assessed using the MWM test. Briefly, mice underwent a 5‐day acquisition phase with four trials per day to locate a hidden platform, followed by a probe trial on day 6 where the platform was removed to assess memory retention. All sessions were tracked using an automated video system.

### 2.8 Hematoxylin & Eosin (HE) Staining

Mice from all experimental groups were deeply anesthetized and transcardially perfused with saline followed by ice‐cold 4% paraformaldehyde (PFA) in phosphate buffer. Brains were post‐fixed in 4% PFA for 24 h, then dehydrated through a graded ethanol series, cleared in xylene, and embedded in paraffin. Coronal sections containing the hippocampus were cut at a thickness of 5 μm using a rotary microtome (Thermo Scientific). After deparaffinization and rehydration, sections were stained with hematoxylin and eosin following standard procedures. Hippocampal structural integrity in the CA1 and CA3 subfields was assessed by a blinded observer using a slide scanner (Pannoramic 250, 3DHistech). This 4% PFA fixative was used consistently for all subsequent histological and immunohistochemical procedures.

### 2.9 Immunofluorescence and Immunohistochemistry

Hippocampal tissue from mice in all experimental groups (including sham, model, and treated groups at both acute (24 h) and chronic (28 days) time points) was homogenized, and proteins were separated by SDS‐PAGE. After transfer to PVDF membranes, blots were incubated with primary antibodies against gamma‐aminobutyric acid type A receptor subunit alpha 1 (GABRA1) and N‐methyl‐D‐aspartate receptor subunit 2B (NR2B) for acute phase samples, or GABRA1, NR2B, sarco/endoplasmic reticulum calcium ATPase 2 (SERCA2), brain‐derived neurotrophic factor (BDNF), phosphorylated protein kinase B (pAKT), and cAMP response element binding protein (CREB) for chronic phase samples, followed by HRP‐conjugated secondary antibodies. Protein bands were visualized using ECL and quantified with Image Lab Software, with β‐actin or GAPDH as loading controls.

### 2.10 In Silico Modeling of Cortical Spreading Depolarization (CSD)

We leveraged a computational model to investigate whether the synergy between Na_V_ blockade and GABA_A_R potentiation extends to the suppression of CSD, a key pathophysiological process in refractory epilepsy [13, 14]. The model comprised a minimal microcircuit of interconnected neurons: a pyramidal glutamatergic neuron and a GABAergic interneuron. Their interactions included: (1) a GABAergic synapse from the interneuron to the pyramidal cell, (2) a glutamatergic synapse from the pyramidal cell to the interneuron, and (3) an excitatory autaptic connection on the pyramidal cell. To simulate an epileptogenic environment predisposed to CSD, we introduced two key modifications to the original model: Constant external depolarizing inputs were applied to both GABAergic interneurons (g_D,i_ = 0.2 mS/cm2) and pyramidal neurons (g_D,e_ = 0.2 mS/cm^2^). The baseline extracellular potassium concentration ([K^+^]out) was elevated to 10 mM to reflect the pathological ionic homeostasis associated with epilepsy [15, 16]. Drug effects were simulated by adjusting specific conductances: Na_V_ channel inhibition was modeled by reducing the maximal sodium conductance (g_Na_) from 100 to 10 mS/cm^2^ in both neuron types. GABA_A_R potentiation was represented by augmenting the chloride conductance (g_Cl_) at GABAergic synapses onto pyramidal neurons from 0.1 to 0.4 mS/cm^2^. Simulations were run to assess the latency and propensity for CSD induction under control and treatment conditions. The source code and original parameters are detailed in the referenced literature [14].

### 2.11 Statistical Analysis

Data are expressed as mean ± standard deviation (SD). Statistical analyses were performed using GraphPad Prism 9.0. Before analysis, the normality of data distribution was assessed using the Shapiro–Wilk test, and the homogeneity of variances was evaluated using Bartlett's test. Group comparisons were conducted using appropriate parametric tests (Student's *t*‐test, one‐way ANOVA with Bonferroni's post hoc test) only when both normality and homogeneity of variance assumptions were satisfied. For datasets that violated these assumptions, the corresponding non‐parametric tests (Mann–Whitney U test or Kruskal‐Wallis test with Dunn's post hoc test) were employed. Categorical variables (e.g., seizure incidence, mortality) were analyzed using the chi‐square test. A *p* < 0.05 was considered statistically significant.

## 3 Results

### 3.1 BETMB Acts as a Dual‐Target Modulator of GABA_A_Rs and Na_V_ Channels

**FIGURE 1:**
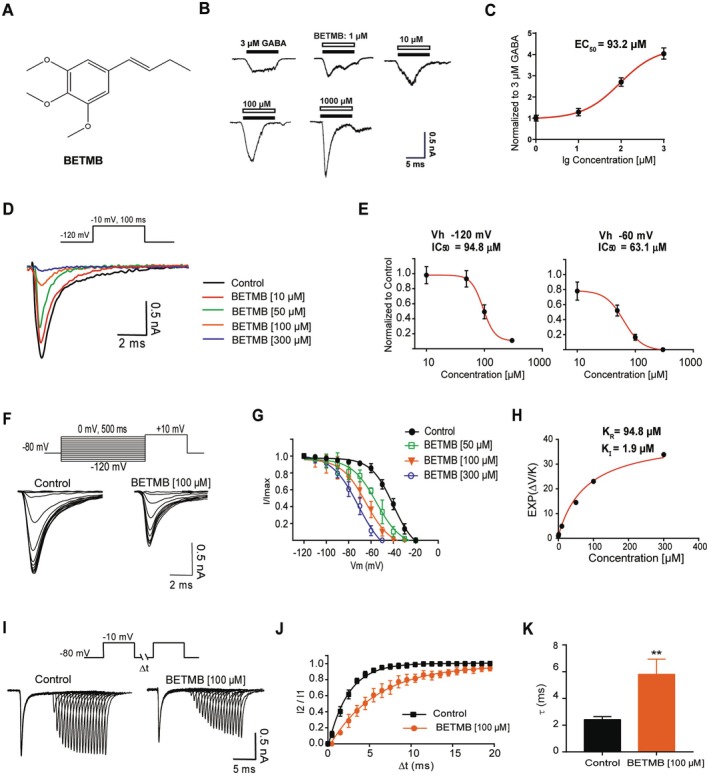
Positive allosteric modulation of GABA_A_Rs and inhibition of Na_V_s by BETMB. (A) Chemical structure of BETMB. (B) Representative current traces induced by 3 μM GABA in the presence of varying concentrations of BETMB. (C) Nonlinear regression analysis based on the Hill equation was performed to calculate the half‐maximal effective concentration (EC_50_) of BETMB for synergistic activation of GABA_A_ receptors (*n* = 3). (D) Representative votage‐gated sodium current traces recorded at BETMB concentrations of 0, 10, 50, 100 and 300 μM BETMB. (E) Nonlinear regression analysis using the Hill equation was carried out to determine the half‐maximal inhibitory concentration (IC_50_) of BETMB on Na_V_s. (F) Representative Na_V_ inactivation currents traces obtained under baseline conditions and at 100 μM BETMB. (G) Single exponential fitting was applied to generate inactivation curves across BETMB concentrations ranging from 0 to 300 μM (H) By incorporating the IC_50_ value (94.8 μM) of BETMB at −120 mV into the analysis, the binding affinity constant for BETMB to the inactivated state of (K_I_) was calculated as 1.9 μM. (I) Representative recovery current traces of sodium channels under control conditions and 100 μM BETMB. (J) Recovery curve of sodium channel activity fitted using a single exponential function. (K) Comparison of the recovery time constant (τ) of sodium channel between the control group and the 100 μM BETMB group (*n* = 6). **p < 0.01 vs. control group.

BETMB (Figure 1A) exhibited no direct agonist activity at GABA_A_ receptors, as application of 500 μM BETMB alone evoked no detectable currents. Co‐application of BETMB (1–1000 μM) with a sub‐saturating concentration of GABA (3 μM) resulted in a concentration‐dependent potentiation of GABA‐evoked currents (Figure 1B). Nonlinear fitting of the concentration‐response relationship yielded an EC_50_ of 93.2 μM and a maximal potentiation of 4.3 ± 0.2‐fold (Figure 1C) (*n* = 3).

Simultaneously, BETMB demonstrated voltage‐dependent inhibition of Na_V_ channels, with IC_50_ values of 94.8 μM at −120 mV (resting state) and 63.1 μM at −60 mV (partially inactivated state), indicating a higher affinity for the partially inactivated state of Na_V_s (Figure 1D,E) (*n* = 6). Analysis of steady‐state inactivation curves revealed a concentration‐dependent hyperpolarizing shift in the half‐inactivation voltage (V_1/2_) (Figure 1F,G) (*n* = 6). BETMB displayed a higher affinity for the inactivated state (K_I_ = 1.9 μM) compared to the resting state (KR = 94.8 μM) (Figure 1H). Furthermore, BETMB (100 μM) significantly slowed the recovery time constant (τ) from inactivation from 2.4 ± 0.2 ms to 5.8 ± 1.1 ms (*n* = 6, *p* < 0.01) (Figure 1I–K).

### 3.2 BETMB Demonstrates Favorable Pharmacokinetics and Brain Distribution

**FIGURE 2:**
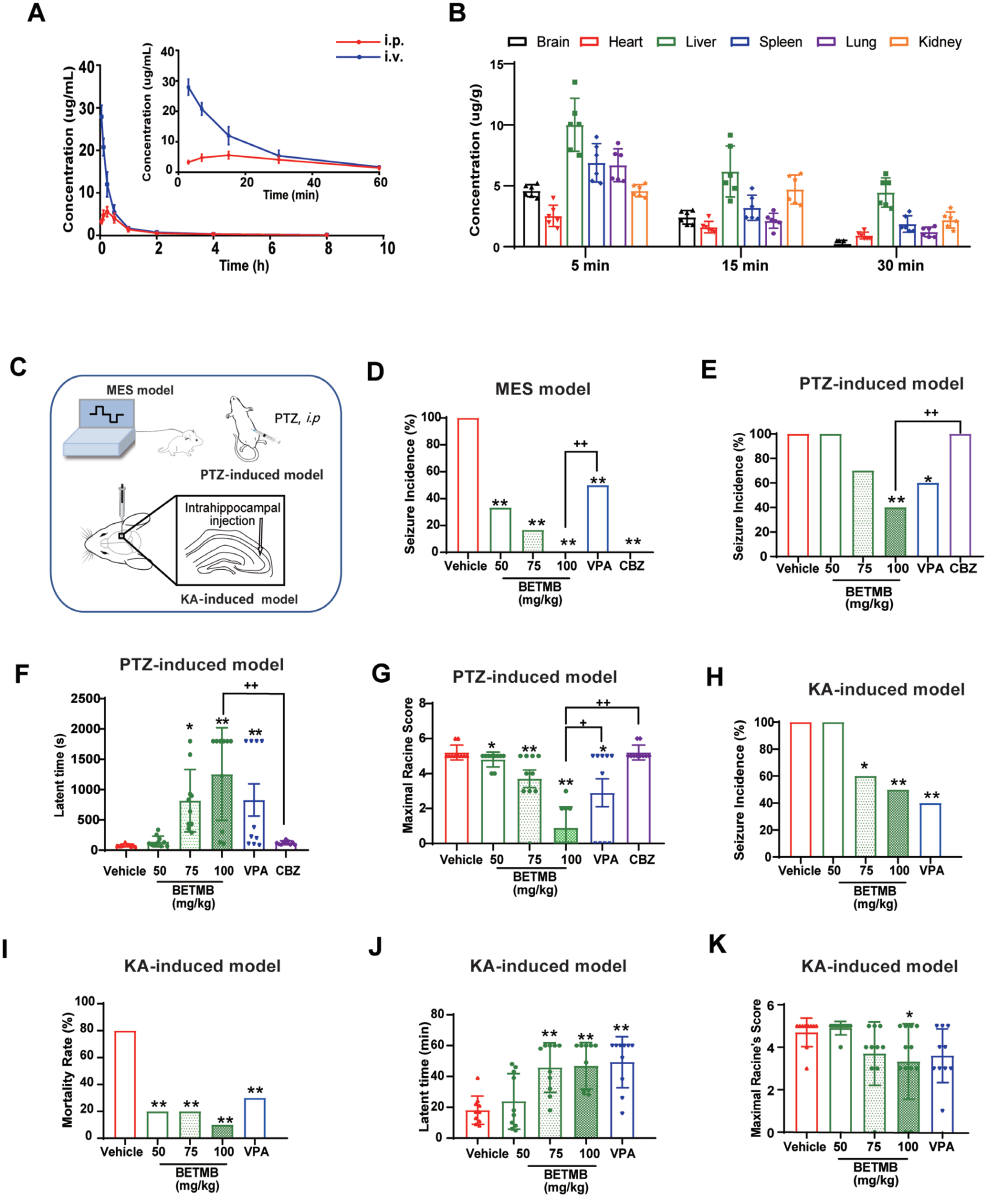
Pharmacokinetic Profile and Broad‐Spectrum Antiepileptic Efficacy of BETMB. (A) Mean plasma concentration‐time curve of BETMB in rats after a single i.v. or i.p. dose of 50 mg/kg (*n* = 6). (B) The concentrations of BETMB in brain, heart, liver, spleen, lung, and kidney tissues were quantified at 5, 15, and 30 min post‐i.p. administration (*n* = 6). (C) Schematic illustration of the experimental seizure induction protocols. (D) Seizure incidence in each group in the MES model (*n* = 10). (E–G) Seizure incidence, latent time (latency to seizure onset), and maximal Racine's score in the PTZ‐induced epilepsy model (*n* = 10). (H–K) Mortality rate, latent time, and maximal Racine's score during the acute phase in the KA model (*n* = 10). *p < 0.05, **p < 0.01 vs. vehicle group; ^*+*^ *p* < 0.05, ^*++*^ *p* < 0.01 vs. BETMB group (100 mg/kg). VPA: Valproic acid; CBZ: Carbamazepine.

BETMB exhibited favorable pharmacokinetic properties and significant brain penetration. Following i.p. administration, it was rapidly absorbed (*T*_max_ = 0.16 h) with an absolute bioavailability of 51.5% (Figure 2A, Table 1). Most notably, BETMB efficiently crossed the blood–brain barrier (BBB), achieving a high brain concentration (4.6 μg/g) within 5 min of i.p. administration (Figure 2B). Tissue distribution analysis at the 5‐min timepoint revealed the following pattern: Liver > Spleen > Lung > Brain ≈ Kidney > Heart. The similar elimination half‐lives after intravenous and i.p. administration suggest route‐independent clearance.

**TABLE 1:** Pharmacokinetic profiles of BETMB after i.v. or i.p. administration (Mean ± SD, *n* = 6).

PK parameters	i.v.	i.p.
AUC (μg·h/mL)	11.56 ± 1.47	5.94 ± 0.58
t_1/2_ (h)	1.35 ± 0.32	1.38 ± 0.32
*T*_max_ (h)	—	0.16 ± 0.02
*C*_max_(μg/mL)	34.38 ± 3.25	5.96 ± 1.08
*F*_*abs*_	100.0%	51.5%

*Note:* Absolute bioavailability was calculated as (AUCi.p./AUCi.v.) × 100%.

Abbreviations: AUC, area under the plasma concentration‐time curve; Cmax, maximum plasma concentration; Fabs, absolute bioavailability; t_1/2_, elimination half‐life; *T*max, time to reach maximum plasma concentration.

### 3.3 BETMB Shows Broad‐Spectrum Efficacy in Acute Seizure Models

BETMB demonstrated broad‐spectrum antiepileptic efficacy in three established seizure models (Figure 2C). In the MES model, BETMB administration dose‐dependently decreased seizure incidence (50 mg/kg: 33.3%, *p* < 0.01; 75 mg/kg: 16.7%, *p* < 0.01; 100 mg/kg: 0.0%, *p* < 0.01 compared to the vehicle group). The 100 mg/kg dose showed significantly superior efficacy to valproic acid (300 mg/kg, i.p.; *p* < 0.01) and provided protection comparable to carbamazepine (100 mg/kg, i.p.) (Figure 2D). Similarly, in the PTZ‐induced model, 100 mg/kg BETMB markedly reduced seizure incidence (40% vs. 100%, *p* < 0.01 compared to the vehicle group), prolonged seizure latency, and lowered maximal Racine scores (*p* < 0.05 vs. valproic acid; *p* < 0.01 vs. carbamazepine) (Figure 2E–G). In the acute phase of the KA‐induced seizure model, compared to the vehicle group, 100 mg/kg BETMB not only decreased seizure incidence (50% vs. 100%, *p* < 0.01) but also extended the latency to severe seizures (*p* < 0.01), attenuated seizure intensity (maximal Racine score: *p* < 0.05), and reduced mortality (10% vs. 80%, *p* < 0.01) (Figure 2H–K).

### 3.4 BETMB Provides Neuroprotection and Modulates Receptor Expression in Acute Phase of KA‐Induced Epilepsy

**FIGURE 3:**
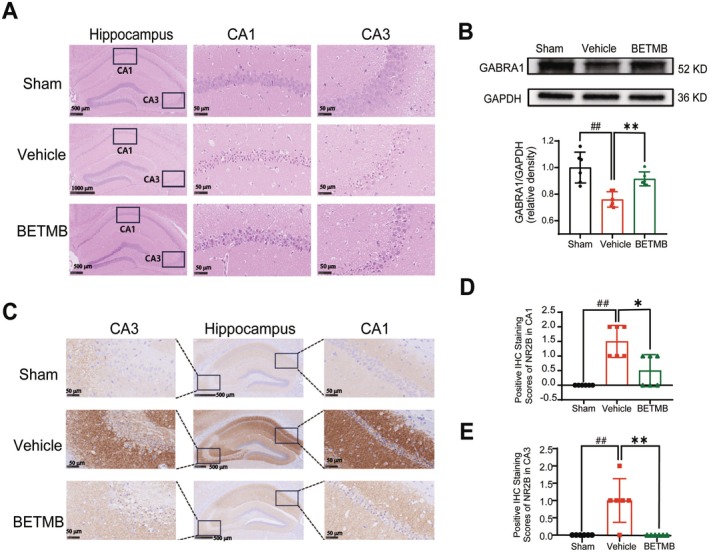
BETMB attenuates hippocampal pathology and modulates GABRA1 and NR2B expression in the acute phase of KA‐induced epilepsy. (A) Representative HE‐stained hippocampal sections from mice in each experimental group during the acute phase of the KA model. (B) Representative western blot bands and quantitative analysis of GABRA1 protein expression levels in the hippocampus during the acute phase of the KA model (*n* = 6). (C–E) Representative immunohistochemical staining images and quantitative analysis of NR2B expression intensity in the CA1 and CA3 subregions of the hippocampus during the acute phase of the KA model (*n* = 6). *p < 0.05, **p < 0.01 vs. vehicle group; ^*##*^ *p* < 0.01 vs. sham group.

HE staining revealed substantial KA‐induced histopathological damage in the hippocampal CA1/CA3 regions during the acute phase, characterized by disorganized neuronal alignment, vacuolar degeneration, nuclear pyknosis, and cytoplasmic lysis. These pathological changes were significantly mitigated by BETMB treatment (Figure 3A). Western blot analysis of whole hippocampal tissue demonstrated that the expression level of the GABRA1 was markedly decreased in vehicle group during the acute phase (*p* < 0.01; *n* = 6). BETMB treatment significantly upregulated GABRA1 expression in the hippocampus (*p* < 0.01; *n* = 6) (Figure 3B). Immunohistochemical analysis of the hippocampal CA1 and CA3 regions showed a significant increase in the expression level of the NR2B during the acute phase of KA *model* (CA1: *p* < 0.01; CA3: *p* < 0.01. *n* = 6). Administration of BETMB significantly downregulated the KA‐induced overexpression of NR2B in both the CA1 (*p* < 0.05) and CA3 (*p* < 0.01) regions (*n* = 6) (Figure 3C–E).

### 3.5 BETMB Mitigates Cognitive Impairments in the Chronic Phase of KA‐Induced Epilepsy

**FIGURE 4:**
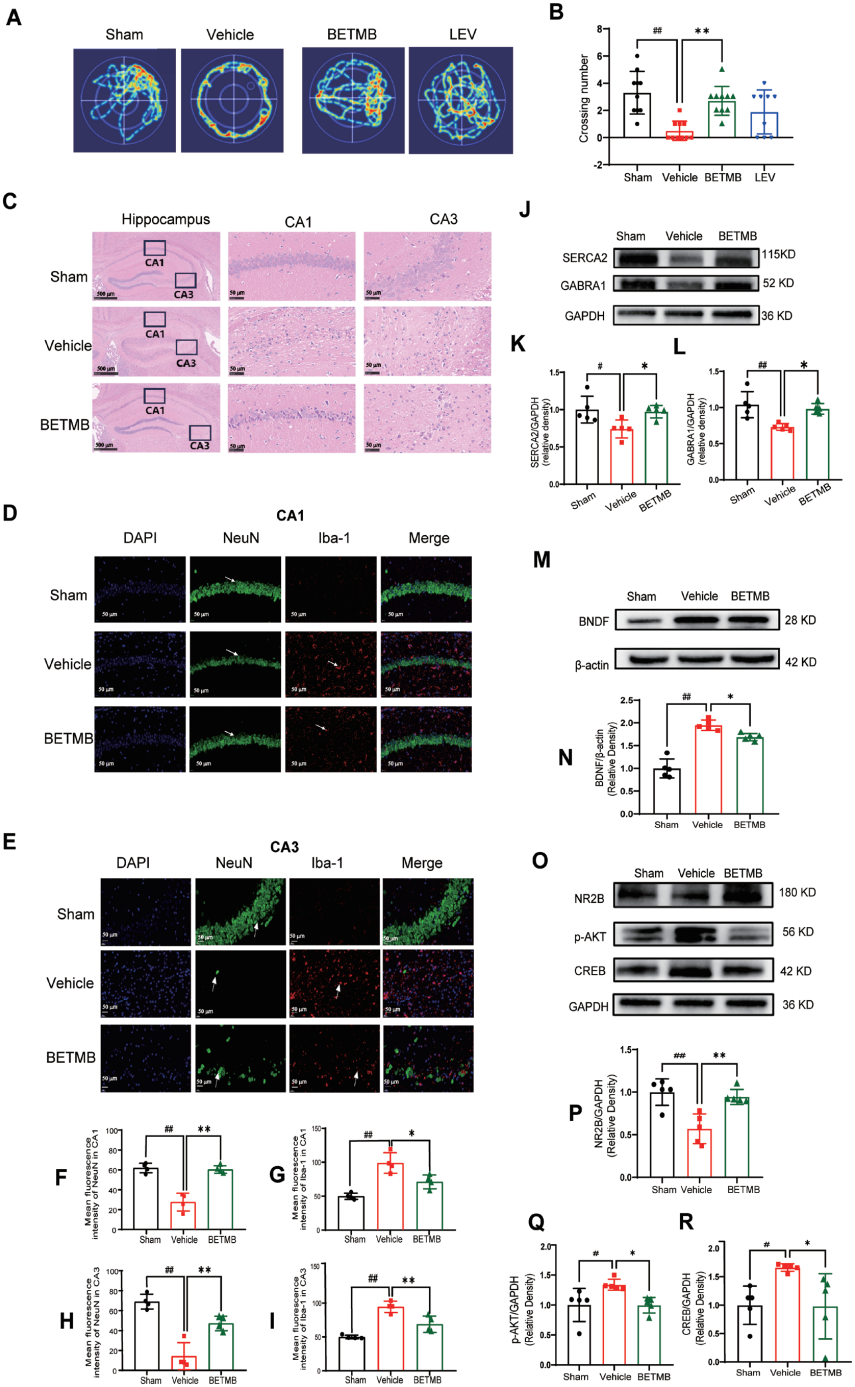
BETMB alleviates cognitive deficits and exerts multi‐target neuroprotection in the chronic phase of KA‐induced epilepsy. (A) Representative trajectory heatmaps from the MWM test probe trial. (B) Statistical analysis of platform crossings in the target quadrant (*n* = 10), showing BETMB significantly improved spatial memory retention. (C) Representative H&E‐stained hippocampal sections, demonstrating that BETMB treatment mitigated KA‐induced structural damage and neuronal disorganization. (D, E) Representative immunofluorescence images and quantitative analysis of NeuN (neuronal marker, green) and Iba‐1 (microglial marker, red) in the CA1 and CA3 regions (*n* = 4). (F − I) Quantitative analysis of NeuN and Iba‐1 fluorescence intensity in the CA1 and CA3 regions (*n* = 4). (J–L) Representative Western blot bands and quantitative analysis of GABRA1 and SERCA2 expression in the hippocampus (*n* = 5). (M, N) Representative Western blot bands and quantitative analysis of BDNF expression in the hippocampus (*n* = 5). (O–R) Representative Western blot bands and quantitative analysis of NR2B, p‐AKT, and CREB expression in the hippocampus (*n* = 5). *p < 0.05, **p < 0.01 vs. vehicle group; ^*#*^ *p* < 0.05, ^*##*^ *p* < 0.01 vs. sham group. LEV: Levetiracetam.

Cognitive function was evaluated in the chronic phase of KA‐induced epilepsy using the MWM test. Mice in the vehicle group exhibited significant spatial memory deficits, demonstrated by markedly fewer platform crossings compared to sham controls (*p* < 0.01, *n* = 10). BETMB treatment substantially improved cognitive performance, as evidenced by significantly increased platform crossings relative to the vehicle group (*p* < 0.01, *n* = 10) (Figure 4A,B). These behavioral results indicate that BETMB effectively ameliorates spatial learning and memory impairments associated with chronic epilepsy.

### 3.6 BETMB Confers Comprehensive Neuroprotection Through Structural Preservation and Multi‐Mechanism Protective Effects in Chronic Epilepsy

Chronic BETMB treatment demonstrated multi‐level neuroprotective effects. HE staining revealed substantial preservation of hippocampal structure, with BETMB treatment significantly attenuating neuronal disorganization and pathological alterations in CA1 and CA3 regions (Figure 4C). IF analysis demonstrated that BETMB effectively reversed neuronal loss and suppressed neuroinflammation. Chronic epilepsy caused substantial neuronal loss (reduced NeuN^+^ intensity) and microglial activation (increased Iba‐1^+^ intensity) in both CA1 and CA3 regions (*p* < 0.01 vs sham controls). BETMB treatment significantly increased NeuN fluorescence intensity (*p* < 0.05 vs vehicle group) and decreased Iba‐1 intensity (*p* < 0.01 vs vehicle group) (*n* = 4; Figure 4D–I). Molecular analysis revealed multi‐mechanism protective effects. BETMB significantly restored GABAergic inhibitory transmission through upregulation of GABRA1, which was down‐regulated in the vehicle group (*p* < 0.01; *n* = 5; Figure 4J,L). The compound also regulated calcium homeostasis by upregulating SERCA2 and NR2B expression (*p* < 0.01, *p* < 0.05; *n* = 5; Figure 4K,P), and effectively suppressed the overactivated BDNF‐pAKT‐CREB signaling pathway (*p* < 0.01; *n* = 5; Figure 4M–O,Q,R).

### 3.7 Dual‐Target Modulation Synergistically Suppresses Epileptiform Discharges In Vitro

**FIGURE 5:**
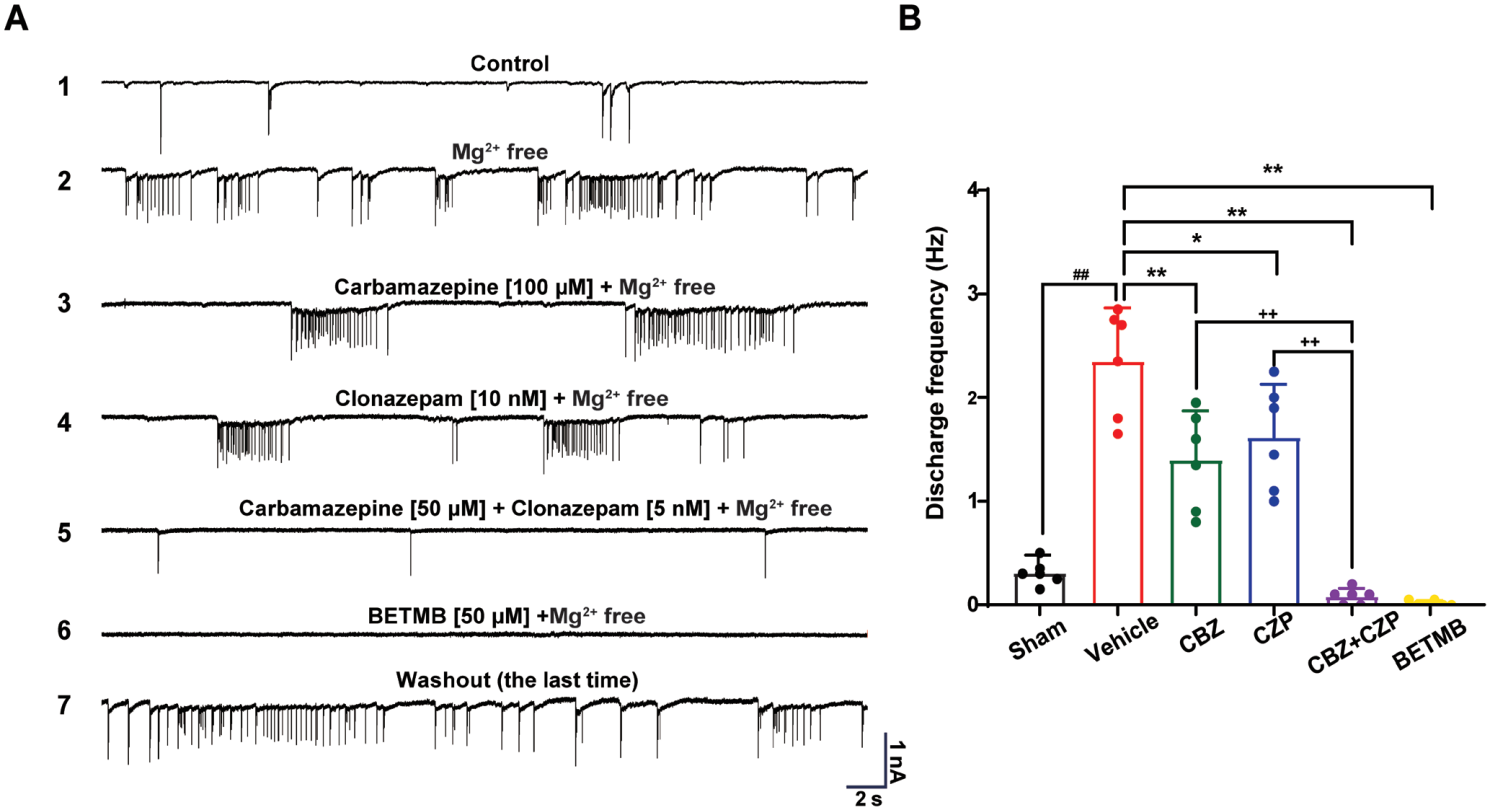
Synergistic inhibitory effects of carbamazepine and clonazepam on neuronal hyperexcitability in the Mg^2+^‐free model. (A) Epileptiform discharge frequencies observed in Mg^2+^‐free vehicle groups. Representative traces (top to bottom): Neuronal firing patterns in (1) sham group, (2) vehicle group, (3) CBZ‐treated group, (4) CZP‐treated group, (5) combination treatment with CBZ + CZP, (6) BETMB‐treated group, and (7) washout phase (final recording). (B) Quantitative analysis of firing frequency (Hz) across experimental groups (*n* = 6). *p < 0.05, **p < 0.01 vs. vehicle; ^*##*^ *p* < 0.01 vs. sham; ^*+*^ *p* < 0.05, ^*++*^ *p* < 0.01 vs. CBZ + CZP combination group. CBZ, carbamazepine; CZP, clonazepam.

In the Mg^2+^‐free extracellular solution‐induced epileptiform discharge model, the neuronal firing frequency significantly increased from 0.31 ± 0.13 Hz (sham) to 2.38 ± 0.45 Hz (vehicle group, *p* < 0.01 vs. control) (Figure 5A,B). Monotherapy with carbamazepine (100 μM) or clonazepam (10 nM) partially attenuated hyperexcitability, reducing firing frequencies to 1.40 ± 0.50 Hz (41.2% inhibition, *p* < 0.01 vs. model) and 1.62 ± 0.55 Hz (31.9% inhibition, *p* < 0.05 vs. model), respectively. Notably, the combination of carbamazepine (50 μM) and clonazepam (5 nM) exhibited supra‐additive efficacy, nearly abolishing discharges to 0.08 ± 0.08 Hz (96.6% inhibition, *p* < 0.01 vs. model; *p* < 0.01 vs. monotherapies). This effect was significantly greater than the theoretical additive inhibition (73.1%). Furthermore, BETMB achieved an inhibition rate of 99.2% (0.02 ± 0.03 Hz, *p* < 0.01 vs. vehicle group).

### 3.8 Dual‐Target Modulation Prevents CSD *in Silico*

**FIGURE 6:**
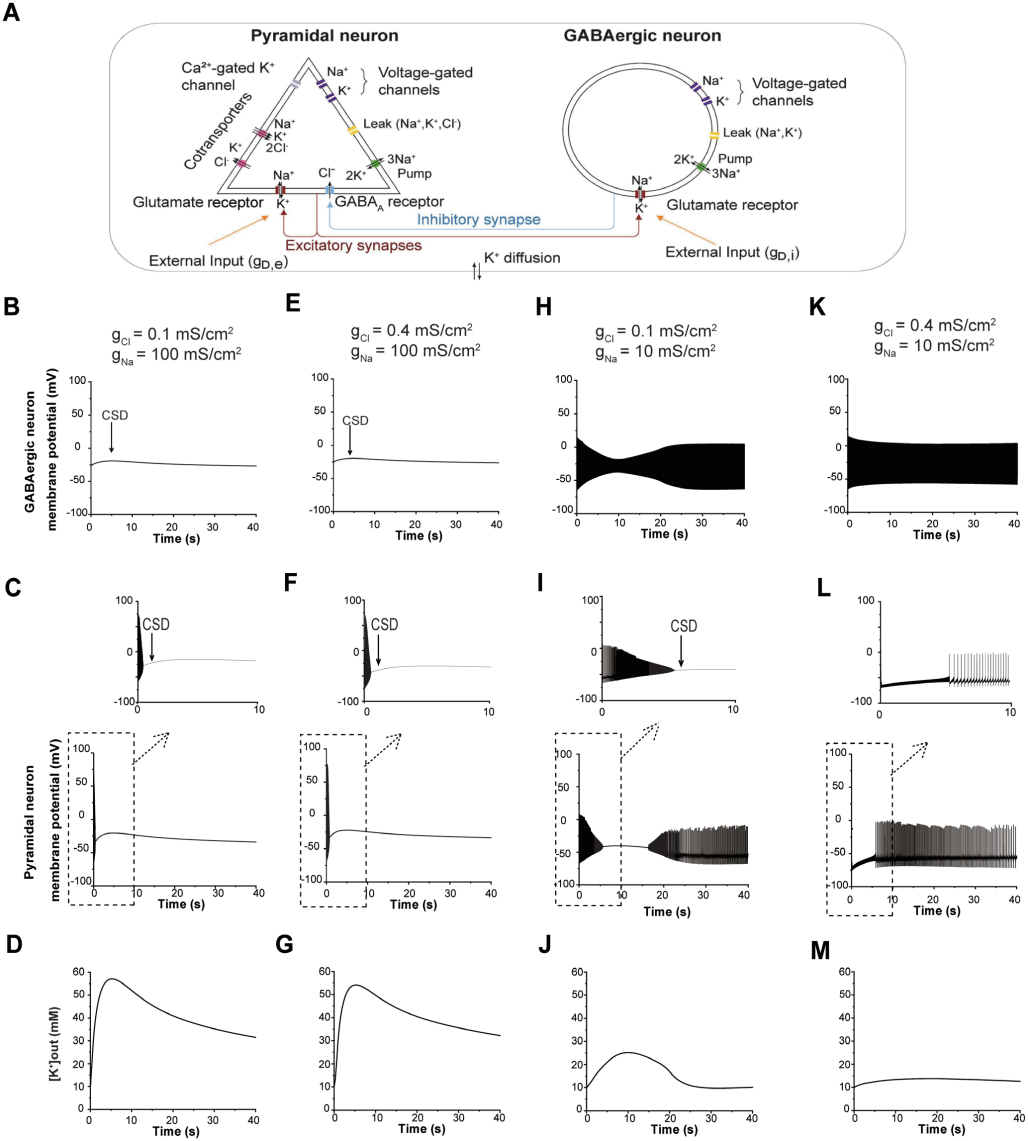
Effects of sodium channel inhibition and/or enhancement of chloride current on CSD Initiation in silico. (A) Schematic diagram of the conductance‐based computational model. (B–D) Simulation results showing the effects of a constant external depolarizing input on GABAergic neuron (gD,i = 0.2 mS/cm^2^) and pyramidal neuron (gD,e = 0.2 mS/cm^2^): Membrane potential of the GABAergic neurons (upper panel), membrane potential of the pyramidal neuron (middle panel), and extracellular K^+^ concentration (lower panel). (E–G) The same simulation with an increased chloride current in pyramidal neuron (g_Cl_ increased from 0.1 mS/cm^2^ to 0.4 mS/cm^2^). (H–J) The same simulation with reduced sodium currents in both GABAergic and pyramidal neurons (g_Na_ decreased from 100 mS/cm^2^ to 10 mS/cm^2^). (K–M) The same simulation combining enhanced chloride current in pyramidal neuron (g_Cl_ increased from 0.1 mS/cm^2^ to 0.4 mS/cm^2^) and reduced sodium currents in both GABAergic and pyramidal neurons (g_Na_ decreased from 100 mS/cm^2^ to 10 mS/cm^2^).

We employed a conductance‐based computational model to simulate neuronal circuits under epileptic conditions (Figure 6A). Under pro‐epileptic conditions with elevated extracellular potassium and sustained depolarization inputs, CSD was consistently triggered within 1 min, characterized by a rapid surge in [K^+^]out to approximately 50 mM (Figure 6B–D). Isolated interventions proved insufficient to block CSD initiation. Enhancing GABAergic transmission through increased chloride conductance showed minimal effect on CSD latency (Figure 6E–G), while selective reduction of sodium currents delayed CSD onset but failed to prevent its eventual development (Figure 6H–J). Notably, the combined strategy of Na_V_ blockade and GABA_A_R potentiation completely suppressed CSD, maintained extracellular potassium homeostasis, and preserved physiological neuronal firing patterns (Figure 6K–M).

## 4 Discussion

BETMB demonstrates favorable drug‐like properties and rapid BBB penetration, supporting its potential as a CNS therapeutic. Pharmacological profiling reveals a dual mechanism of action: positive allosteric modulation of GABA_A_ receptors and state‐dependent inhibition of Na_V_s. This selective targeting suppresses neuronal hyperexcitability while preserving normal signaling, underpinning a promising therapeutic window.

In vivo, BETMB exhibits broad‐spectrum anti‐seizure efficacy across multiple epilepsy models. The therapeutic doses provide effective brain exposure while maintaining a wide safety margin, with an LD_50_ = 745 mg/kg, which is significantly higher than the therapeutic range (Table S1), confirming a favorable efficacy‐safety profile. In the chronic KA model, which replicates key features of human temporal lobe epilepsy [17], BETMB treatment not only reduced spontaneous seizures but also improved cognitive function in MWM tests. These therapeutic benefits were supported by BETMB's ability to mitigate neuropathological damage and neuronal loss. Beyond its direct channel/receptor modulation, BETMB exerts comprehensive effects on downstream protein networks, consistent with established epilepsy pathophysiology. It upregulates GABRA1, a key subunit present in approximately 60% of GABA_A_Rs, a change consistent with bolstering inhibitory signaling [18, 19]. Furthermore, BETMB bidirectionally regulates the NMDA receptor NR2B subunit, which plays a critical role in epileptogenesis [20, 21]. It reduces NR2B overexpression in the acute phase, aligning with studies showing that NR2B antagonists suppress excitability and restores its expression in the chronic phase to support cognitive function, countering the memory impairments associated with low NR2B levels [22, 23, 24, 25, 26]. Concurrently, BETMB upregulates SERCA2, which may contribute to normalizing calcium homeostasis [27, 28, 29]. Finally, it normalizes the overactive BDNF‐pAKT‐CREB signaling pathway, a key driver of neuronal excitability and epileptogenesis that is upregulated in epilepsy models [30, 31, 32, 33], and whose inhibition reduces seizure severity [34, 35]. This integrated multi‐level modulation enables BETMB to simultaneously enhance inhibitory tone, reduce neuronal hyperexcitability, maintain calcium balance, and restore neurotrophic signaling.

The therapeutic superiority of this GABA_A_Rs‐Na_V_ dual‐target approach became evident when we probed the neuroelectrophysiological basis of the synergy. By modifying a previously described method through brief exposure of cultured hippocampal neurons to Mg^2+^‐free medium, we successfully replicated the dual excitatory‐inhibitory dysregulation that characterizes refractory epilepsy [36]. Specifically, Mg^2+^ deprivation‐induced hyperactivation of NMDA receptors triggered Ca^2+^‐dependent slow depolarization and hypersynchronous neuronal discharges. Concurrently, impaired GABAergic inhibition further exacerbated this dysregulation [37]. Moreover, these neurons are capable of forming fully functional synapses and networks, as evidenced by synaptic currents and Mg^2+^‐free‐induced hyperactivity model [36, 38]. Our results demonstrate that combined subtherapeutic doses of a Na_V_ blocker and a GABA_A_R agonist completely abolish hypersynchronous neuronal discharges, demonstrating a synergistic interaction between the two targets.

We then hypothesized that this GABA_A_R‐Na_V_ synergy represents a universal mechanism for stabilizing neuronal networks. To test this, we extended our investigation along the excitability continuum to CSD, a pathophysiological wave strongly implicated in the clinical context of refractory epilepsy. CSD is characterized by a propagating wave of massive neuronal and glial depolarization, followed by a prolonged suppression of neural activity [39, 40]. Critically, a wealth of evidence underscores a close, bidirectional association between CSD and seizures, particularly in refractory cases [41, 42, 43]. Experimental models have demonstrated that seizures can trigger repetitive CSD events, and conversely, CSD can precede and facilitate ictal activity. Most significantly, recent clinical studies have revealed that all patients with refractory epilepsy exhibited CSD, with varying time lags before the onset of seizures [44], suggesting it may disrupt the excitation‐inhibition balance, increase neuronal excitability and facilitate seizure activity [45, 46]. A key pathophysiological link between seizures and CSD is the dysregulation of extracellular potassium. During intense epileptiform activity, [K^+^]out can rise from a baseline of ~3 mM to levels of 10–12 mM, and further surge to 50 mM or higher at the core of a propagating CSD wave, creating a self‐reinforcing cycle of depolarization [47, 48]. This continuum of [K^+^]out elevation underpins the shared excitotoxicity mechanism. Therefore, we proposed that an effective therapy for refractory epilepsy must address not only seizures but also this CSD. Our computational results, which incorporated this pathophysiological [K^+^]out dynamic, demonstrate that only combined GABA_A_R potentiation and Na_V_ inhibition prevents cortical spreading depression and maintains K^+^ homeostasis, directly disrupting this critical excitability loop.

Epilepsy is not merely a disorder of isolated hyperexcitability but a systemic failure of network stability, characterized by a vicious cycle of disrupted ion homeostasis, impaired inhibitory neurotransmission, and runaway excitatory depolarization. Single‐target agents, such as a Na_V_ blocker or a GABA_A_R modulator alone, operate on a single axis of this dysregulated network. While partially effective, they are inherently limited because the pathology simultaneously undermines their action elsewhere. For instance, a non‐selective Na_V_ blocker suppresses both principal glutamatergic neurons and GABAergic interneurons, impairing the network's intrinsic inhibitory capacity. This is critically exemplified by the pathophysiology of SCN1A‐deficient epilepsies like Dravet syndrome, where the Na_V_1.1, preferentially enriched in GABAergic interneurons, is already compromised [49, 50]. Administration of a broad‐spectrum Na_V_ blocker like carbamazepine further cripples the firing of these already impaired inhibitory neurons, leading to a net loss of inhibition that can exacerbate seizures [51, 52]. For GABA_A_R agonists, while GABAergic interneurons possess intrinsic self‐protection mechanisms (e.g., receptor diversity, specific circuit integration) that prevent their own over‐inhibition, chronic enhancement of GABA_A_R transmission with a single‐target agent faces its own fundamental limitation: the development of tolerance. This arises from compensatory neuroadaptations, including receptor internalization, altered subunit composition that reduces drug sensitivity, and a collapse of the chloride ion gradient that can paradoxically convert GABAergic inhibition into excitation [53]. In stark contrast, the Na_V_‐GABA_A_R dual‐target strategy embodied by BETMB is uniquely positioned to address the inherent limitations of both single‐target approaches. First, while Na_V_ blockade may impair GABAergic interneuron function, simultaneous GABA_A_R potentiation directly compensates for this deficit by enhancing inhibitory transmission. Second, the state‐dependent Na_V_ blockade preferentially suppresses hyperactive neurons, reducing the workload of interneurons in suppressing nervous system hyperexcitability. This reduction in demand has the potential to mitigate the neuroadaptation leading to tolerance, allowing for sustained therapeutic effects even with reduced GABA receptor involvement.

In conclusion, our research goes beyond merely introducing BETMB as a novel dual‐target anti‐seizure compound. For the first time, we present electrophysiological evidence demonstrating the synergy between GABA_A_R and Na_V_ in suppressing hyperactivity. Through computational modeling, we further illustrate that this synergistic mechanism spans across the unified neuronal dynamic continuum to prevent CSD. These findings construct a comprehensive framework for comprehending and treating a range of hyperexcitable brain states via the coordinated modulation of inhibitory and excitatory drives. This framework offers novel therapeutic strategies for refractory epilepsy and other conditions associated with spreading depolarization, including migraine, stroke, and traumatic brain injury.

We acknowledge several limitations in the present study. First, the precise molecular target profile remains incompletely resolved, as we have not employed recombinant systems to determine BETMB's selectivity for specific GABA_A_ receptor subtypes or for individual Na_V_ isoforms. Second, the study lacks epileptic activity data in brain tissue or in the brains of epileptic animals, which would bridge cellular mechanisms to network‐level dysfunction. Furthermore, as noted, the individual versus synergistic contributions of its dual‐target modulation to the observed outcomes remain unclear, and the absence of a sham group treated with BETMB alone in the chronic model limits the mechanistic interpretation of the molecular rescue effects. We believe that more in‐depth mechanistic investigations will further refine our findings.

## Author Contributions

Di Zhang: Conceptualization; Methodology; Formal analysis; Writing, original draft; Writing, review and editing. Kai Li: Investigation; Writing, review and editing. Yingying Zhang: Software. Yao Nie: Investigation. Yue Li: Data curation. Rui Li: validation. Xin Wang: Visualization. Shengjun Mao: Funding acquisition; Project administration; Resources; Supervision; Writing, review and editing.

## Funding

The authors have nothing to report.

## Conflicts of Interest

The authors declare no conflicts of interest.

## Supporting information


**Table S1:** LD_50_ of BETMB.

## Data Availability

The data that support the findings of this study are available on request from the corresponding author. The data are not publicly available due to privacy or ethical restrictions.
