# Supplementary material for: BETMB: A Dual‐Target Compound for Synergistic Suppression of Neuronal Hyperexcitability in Refractory Epilepsy via Concurrent Modulation of Nav Channels and GABAA Receptors
Source: CNS Neurosci Ther. 2026 Jan 23;32(1):e70766. doi: 10.1002/cns.70766 (PMC12828663; doi:10.1002/cns.70766)
Supplement: Supplementary file 1 — Table S1: LD50 of BETMB. [file CNS-32-e70766-s001.pdf]

Supplementary Materials for

BETMB: A Dual-Target Compound for Synergistic Suppression of Neuronal Hyperexcitability in Refractory Epilepsy via Concurrent Modulation of Nav Channels and $GABA_A$ Receptors

Di Zhang, Kai Li, Yingying Zhang, Rui Li, Xin Wang, Yao Nie, Shengjun Mao*

Key Laboratory of Drug-Targeting and Drug Delivery System of the Education Ministry and Sichuan Province, Sichuan Engineering Laboratory for Plant-Sourced Drug and Sichuan Research Center for Drug Precision Industrial Technology, West China School of Pharmacy, Sichuan University, Chengdu, 610041, China

***Corresponding author**

E-mail address: xinba789@163.com (Shengjun Mao).

This file includes：

Supplementary Methods

Table S1

Supplementary Methods

Patch-clamp recordings

All electrophysiological experiments were conducted in the whole-cell patch-clamp configuration under voltage-clamp mode for current recordings. Recording electrodes, with resistances ranging from 3 to 8 MΩ, were fabricated from borosilicate glass capillaries (outer diameter: 1.5 mm; inner diameter: 0.86 mm) using a Sutter Instruments P-97 micropipette puller. Current acquisition and voltage command execution were controlled by a Heka EPC-10 amplifier operated via Patchmaster software. Series resistance (Rs) was compensated using the amplifier's automatic compensation circuitry. Typically, 70-85% compensation was applied with a lag setting of 10 μs to remain below a final effective Rs of 10 MΩ. The liquid junction potential (LJP) was calculated using the PatchMaster software and compensated for during the experimental procedure. Post-experiment cell viability was confirmed by a sustained membrane resistance (Rm) exceeding 500 MΩ. Capacitive artifacts and leak currents were minimized through online P/4 subtraction protocols, while signals were filtered using a 4-pole Bessel low-pass analog filter at 5 kHz prior to digitization at a sampling rate of 50 kHz.

$GABA_A$ receptor currents were isolated using an extracellular solution containing (in mM): 140 NaCl, 5 CsCl, 2 $CaCl_2$, 1 $MgCl_2$, 5 HEPES, and 10 D-glucose (pH = 7.4, adjusted with NaOH; osmolarity = 305 mOsm/L) and a pipette solution containing (in mM): 130 CsCl, 0.1 $CaCl_2$, 10 HEPES, 5 EGTA, 5 $MgCl_2$, and 2 Na_2-ATP (pH = 7.2, adjusted with CsOH; osmolarity = 295 mOsm/L). Na_V currents were characterized using an extracellular solution containing (in mM): 140 NaCl, 5 CsCl, 2 $CaCl_2$, 1 $MgCl_2$, 20 TEA-Cl, 0.2 $CdCl_2$, 10 HEPES, 10 D-Glucose and 4-aminopyridine (pH = 7.4, adjusted with NaOH; osmolarity = 315 mOsm/L), and a pipette solution containing (in mM): 10 NaCl, 100 CsCl, 10 HEPES, 11 EGTA, 5 $MgCl_2$, 10 TEA-Cl and 2 Na_2-ATP (pH = 7.2, adjusted with CsOH; osmolarity = 300 mOsm/L). BETMB was dissolved in DMSO to prepare a 500 mM stock solution, which was subsequently diluted in the respective extracellular solution to the final working concentration (maximum DMSO concentration ≤ 0.2%, which was confirmed to have no effect on its own). All agents were applied via a gravity-fed bath perfusion system controlled by an eight-valve controller (Warner Instruments) to ensure rapid and complete solution exchange.

For $GABA_A$ receptor experiments, neurons were voltage-clamped at -80 mV. A full concentration-response curve for BETMB was constructed by pre-applying each concentration for 3 minutes prior to its co-application with GABA (3 μM). A 15-minute washout period in drug-free extracellular solution was implemented between each drug application to ensure complete recovery from the previous effect. This protocol was performed on the same cell (n = 3). For Na_V channel experiments, BETMB was pre-applied for 3 minutes before initiating the voltage-protocol stimuli.The voltage protocols for characterizing Na_V channels were as follows. To determine the voltage-dependent inhibitory potency (IC_{50}), cells were held at either -120 mV (resting state) or -60 mV (partially inactivated state), and a series of 100-ms test pulses to -10 mV were applied to elicit Na^+ currents in the absence and presence of increasing concentrations of BETMB. For other protocols, neurons were voltage-clamped at a holding potential of -80 mV. To assess steady-state inactivation, a standard two-pulse protocol was used: cells were conditioned by a series of 500-ms pre-pulses ranging from -120 mV to 0 mV in 10-mV increments, each immediately followed by a 100-ms test pulse to +10 mV. The peak test-pulse current (I) was normalized to the maximum current obtained after the -120 mV pre-pulse (I_{max}). To analyze recovery from inactivation, a double-pulse protocol was employed: a 20-ms conditioning pulse to -10 mV inactivated the channels, followed by a return to -80 mV for a variable recovery interval (Δt, from 0.5 ms to 19.5 ms in 1-ms increments) prior to the delivery of an identical test pulse; the recovery ratio (I_2/I_1) was then plotted against Δt. Quantitative analyses of BETMB on $GABA_A$Rs and Na_Vs included the following methodologies: 1) Dose-response fitting using the Hill equation to evaluate the synergistic effect on $GABA_A$Rs or inhibitory effect on Na_Vs: $Y= Bottom + (Top-Bottom)/[1 + (D/XC_{50})^H]$, where Y denotes the activation or inhibition response, *Bottom* is the minimum response, *Top* is the maximum response, D is the drug concentration, XC_{50} is the half-maximal effective concentration (EC_{50} or IC_{50}) and H is the Hill coefficient. 2) Inactivation curve fitting using the Boltzmann equation: $I/I_{max} = 1 / [1 + exp ((V - V_{1/2})/k)]$, where I is the current, I_{max} is the maximum current, V is the applied voltage, $V_{1/2}$ is the half-inactivation voltage, and k is the slope factor. 3) Calculation of compound affinity for Na_Vs based on the shift in inactivation voltage: $exp(\Delta V_{1/2}/k) = (1+D/K_I)/(1+D/K_R)$, where $\Delta V_{1/2}$ represents the change in the half-inactivation

voltage, D is the drug concentration, k represents the slope factor, K_I is the affinity of the compound for the inactivated state of the Na_V, and K_R is the affinity for the resting state. 4) Recovery curve fitting using a single exponential equation: $I = A*exp(t/\tau) + C$, where I is the current, A and C are constants, t is the recovery time, *and* τ is the recovery time constant.

For assessments of network hyperactivity and hypersynchrony, the Mg^{2+}-free extracellular solution was composed of (in mM): 140 NaCl, 2 $CaCl_2$, 5 KCl, 10 HEPES, and 10 D-glucose (pH = 7.4, adjusted with NaOH; osmolarity = 300 mOsm/L). The Mg^{2+}-containing solution was prepared by adding 1 mM $MgCl_2$ to the Mg^{2+}-free solution. The pipette solution consisted of (in mM): 10 NaCl, 100 KCl, 10 HEPES, 11 EGTA, 5 $MgCl_2$, and 2 Na_2-ATP (pH = 7.2, adjusted with KOH; osmolarity = 295 mOsm/L). Synaptic currents were continuously recorded in the same neuron under voltage-clamp at a holding potential of -60 mV. The protocol began with a 5-minute baseline perfusion using the Mg^{2+}-containing solution, followed by induction of epileptiform network activity via 5-minute perfusion with the Mg^{2+}-free medium. Subsequently, antiepileptic compounds, carbamazepine (100 μM), clonazepam (10 nM), a combination of carbamazepine (50 μM) and clonazepam (5 nM), or BETMB (50 μM), were sequentially administered for 5-minute intervals to evaluate their effects on suppressing the synchronous synaptic activity. Each compound application was separated by a 10-minute Mg^{2+}-free washout period to eliminate residual drug effects. Post-experiment cell viability was confirmed by robust neuronal responses to Mg^{2+}-free stimulation. The synaptic currents were recorded in six interneurons surrounded by pyramidal neurons (n = 6). Interneurons were selected for recording based on their morphological characteristics (smaller soma size, high branching complexity). Based on the primary pathophysiology of the low-Mg^{2+} model (i.e., the induction of hyperexcitability through enhanced glutamatergic neurotransmission) coupled with the experimental recording conditions (voltage-clamped at a holding potential of -60 mV), the large-amplitude (about 1 nA), synchronous synaptic events recorded are predominantly network-driven excitatory postsynaptic currents (EPSCs). The hyperexcitability of the neuronal network was assessed by measuring the frequency of the large-amplitude synaptic currents. These currents reflect synchronous population discharges, and their frequency is reported as the network discharge frequency.

Animal epilepsy models

Male C57BL/6 mice (weighing 18–22 g, 6–8 weeks) were utilized for the establishment of seizure models. For MES, PTZ, and KA-induced acute seizure models, mice were randomly divided into six groups: (1) an epilepsy-induced model group receiving normal saline without therapeutic intervention; (2–4) three BETMB-treated groups (50, 75, and 100 mg/kg, i.p.); and (5–6) positive control groups administered carbamazepine (100 mg/kg, i.p.) or valproic acid (300 mg/kg, i.p.). The positive control drugs were prepared as follows: valproic acid and levetiracetam were dissolved in normal saline, while carbamazepine was dissolved in a propylene glycol/ethanol/normal saline mixture (5:2:3, v/v).

Seizure induction was performed using three established models. Seizure severity was evaluated according to the Racine scale. In the MES test, a 150 V, 50 Hz, 0.2-s electrical stimulus was delivered via auricular electrodes moistened with normal saline using a YLS-9A simulator. Drugs or normal saline were administered 5 min prior to seizure induction, and hindlimb tonic extension was recorded as the endpoint. In the PTZ model, seizures were induced by intraperitoneal injection of PTZ (70 mg/kg in normal saline) 3 min after drug or normal saline administration. Seizure severity was evaluated with the maximum Racine scale and latency to Racine scale $\geq$ 4 convulsions documented within 30 min (latency = 1800 s if no convulsions occurred). In the KA-induced acute model, mice received stereotaxic intrahippocampal injection of KA (1 mg/mL in normal saline, 15 μg/ kg; coordinates: AP -2.00 mm, L -2.50 mm, DV 2.00 mm relative to bregma) under 4% isoflurane anesthesia. Postoperative wounds were disinfected with povidone-iodine and treated with erythromycin ointment for infection prevention. Drugs or normal saline were administered 10 min post-KA injection, and seizure parameters (latency to Racine scale $\geq$ 4 seizures, maximum Racine score, mortality) were monitored for 60 min (latency = 60 min if no convulsions occurred). For chronic epilepsy studies, mice exhibiting sustained Racine scale $\geq$ 4 seizures for $\geq$1 h following KA induction were randomly divided into four groups: Sham (normal saline), Vehicle (blank micellar solution), BETMB (75 mg/kg, i.p. daily), and levetiracetam (400 mg/kg, i.p. daily). The first therapeutic dose was administered 1 hour after the onset of seizures (i.e., upon meeting the inclusion criterion of sustained seizure activity), followed

by once-daily dosing thereafter. After 20 consecutive days of treatment, cognitive function was assessed using the Morris water maze (MWM) test.

Acute toxicity study

Using the Bliss method, the LD_{50} value was determined to be 745 mg/kg, with a 95% confidence interval ranging from 664 to 841 mg/kg.

Supplementary Table

Table S1： LD_{50} of BETMB

Group	Administration Dose (g/kg)	Dose Logarithm (x)	Number of Animals		Number of Deaths		Total (Deaths/Total Animals)	Mortality (p)
			♀	♂	♀	♂		
1	0.40	-0.398	5	5	0	0	0/10	0
2	0.48	-0.319	5	5	1	0	1/10	0.1
3	0.57	-0.244	5	5	1	1	2/10	0.2
4	0.69	-0.161	5	5	2	3	4/10	0.4
5	0.83	-0.081	5	5	4	3	6/10	0.6
6	1.00	0.000	5	5	4	5	8/10	0.8
7	1.20	0.079	5	5	5	5	10/10	1
8	0	-	5	5	0	0	0	0
